# The Influence of the Trainer’s Social Behaviors on the Resilience, Anxiety, Stress, Depression and Eating Habits of Athletes

**DOI:** 10.3390/nu12082405

**Published:** 2020-08-11

**Authors:** Rubén Trigueros, Isabel Mercader, Jerónimo J. González-Bernal, José M. Aguilar-Parra, Josefa González-Santos, Noelia Navarro-Gómez, Raúl Soto-Cámara

**Affiliations:** 1 Department of Psychology, Hum-878 Research Team, Health Research Centre, University of Almeria, 04120 Almeria, Spain; rtr088@ual.es (R.T.); nng777@ual.es (N.N.-G.); 2 Department of Psychology, University of Burgos, 09001 Burgos, Spain; jejavier@ubu.es (J.J.G.-B.); mjgonzalez@ubu.es (J.G.-S.); rscamara@ubu.es (R.S.-C.)

**Keywords:** social behaviour, resilience, sport, mental health, nutrition

## Abstract

During their sporting lives, athletes must face multiple difficulties that can have consequences for their mental health and changes in their eating patterns. Therefore, the present study aims to analyze how social skills of the trainer influence the coping capacity, psychological well-being, and eating habits of the athlete, elements that are key to achieving success during competition. This study involved 1547 athletes and 127 trainer. In order to achieve the objective, the mean, standard deviation, bivariate correlations, reliability analysis and a structural equation model were analysed. The results showed that prosocial behaviours were positively related to resilience, while antisocial behaviours were negatively related. Resilience was negatively related to anxiety, stress and depression. Finally, anxiety, stress and depression were negatively related to healthy eating and positively related to unhealthy eating. These results highlight the importance of creating a positive social climate to develop coping strategies that promote mental health and healthy eating habits of athletes.

## 1. Introduction

In a highly demanding sporting context, the figure of the trainer is essential given his/her influence on athletes [1]. Thus, the trainer’s function is to promote the physical, psychological and social development of their athletes to increase their performance in order to achieve individual and/or team goals [2]. However, the influence that the trainer exerts is not limited only to the sports context, but must understand and pay attention to the psychological, physical and social well-being and habits of each sportsperson [3]. Despite this, few studies have examined the relationship between the social behavior of the trainer on the management of stress during competition by athletes [4] and the consequences on their dietary behaviors [5]. In this sense, several studies give balanced diets a significant relationship with sports performance [6,7]. Therefore, this study aims to analyze how social behaviors of trainers influence the coping capacity, psychological well-being, and eating habits of the athlete, elements that are key to achieving achievements during competition.

### 1.1. Role of the Trainer

Trainers are a reference model for athletes who influence their psychosocial growth [8]. Therefore, in each of the trainer-athlete interactions there is an opportunity for the trainer to transmit positive values, help the development of self-efficacy or increase the self-esteem of the athlete [1]. However, the opposite can happen that the trainer-athlete interaction causes a loss of self-efficacy and self-esteem of the athlete and the transmission of negative values. Therefore, the trainer may cause an increase or decrease in the athlete’s psychological and social well-being.

The influence of the trainer during training has been studied from two points of two opposing aspects, prosocial versus antisocial behaviors [9,10]. The first is related to the promotion of self-concept, intrinsic motivation and personal initiative, making use of minimal contingencies, which are linked to prosocial skills [9,10]. On the contrary, antisocial behaviors is related to the use of coercive means, threats and insults, the loss of personal initiative and controlled motivation, being linked to antisocial skills.

Studies to date suggest that prosocial behaviors is related to the satisfaction of psychological needs, motivation and resilience [2]. Meanwhile, antisocial behaviors are positively related to frustration of psychological needs, controlled motivation, anxiety and sports withdrawal. Nevertheless, several current studies have taken into account the athletes’ perception of their trainer [11]. However, studies such as the one conducted by Haerens, Aelterman, Vansteenkiste, Soenens & van Petegem [12], showed that sometimes the individual’s perception of the teacher/trainer differs from their own perception. Therefore, this study aims to analyze how the trainer’s self-perception influences the ability to overcome stressful events that generate maladaptive behavior in athletes. Hence, this is one of the first studies to analyze these relationships.

### 1.2. Resilience

During the sporting life of any athlete, they have to face multiple stressors (e.g., injuries, defeats, team changes, loss of the championship) that can negatively influence their psychological well-being and affect their life habits [13]. However, every athlete has a number of internal psychological mechanisms to cope with these negative experiences, making them less vulnerable to stressful events [14].

The meta-theory of resilience [15] describes resilience as a mechanism that favours the recovery of optimal levels of functioning and competence (homeostasis) of the subject when faced with a potentially stressful event. However, there are a number of limitations that this meta-theory does not address, as it considers a single event to be in opposition to the individual [16]. On the other hand, this model also does not address how emotions exert a protective character over the behavior of individuals [17], since in different studies athletes value certain emotions as mechanisms that help to overcome stressors [18]. In this way, Fletcher and Sarkar [19] reconceptualized resilience, adapting it to the sports context, as the mechanism that allows the athlete to face a potentially stressful event. Stressors are perceived by athletes as opportunities for growth, development and mastery, that is, they evaluate these situations as a motivating challenge and not as a threat. Thus, those subjects who make this positive assessment of risk or adversity, revolves around the possession and presence of a series of psychological factors inside and outside the individual that, in balance, will lead to optimal sports performance [20].

The main areas that have studied resilience have been in the health field [21], the laboral field [22], the military [23] and recently in the academic field [24]. Studies on resilience highlight the positive effect it has on overcoming stressors and stressful situations, such as sporting failure [25] and injuries [26]. In addition, some studies have highlighted the positive effect that resilience has on athletes’ internal motivation [27], emotional intelligence [28] and psychological well-being [29]. Despite the importance of existing studies, this research has focused on analysing the influence of resilience in relation to psychological or emotional variables. There have been few studies that have attempted to analyse the relationship between resilience and each of the most disabling psychological disorders frequently encountered by athletes.

### 1.3. Anxiety, Stress and Depression

Sometimes the adverse circumstances, or the accumulation of them, cause the athlete to burnout on an emotional level, which can lead to a decrease in sporting performance [30]. This emotional burnout can be due to anxiety, which is characterized by the presence of feelings of distress, uncertainty and tension that arise as a result of any transcendental situation or want to anticipate a real or imaginary threat causing a lack of control of the behavior and thoughts of the athlete [31]. However, during periods of competition, athletes may show a greater propensity, before or during the competition, for anxiety, which is conceptualized as a specific personality trait in the face of that situation of the athlete [32]. Different studies in the field of sport have shown that anxiety is negatively related to sports performance [33], concentration [34], social relations [35] and self-confidence [36]. In contrast, anxiety has been positively related to stress [37], amotivation [38], dropout [39], depression [40] and mental rumination [34].

In addition, the athlete must deal with social and internal pressure and pressure to achieve goals [34]. This pressure can put a psychological burden on the athlete, exhausting his/her reserves and causing his/her inability to react and adapt to the demands of sport, at this point, is what is known as stress [41]. On the other hand, depression is a mental illness characterized by a deep mood disorder. Depression has as its main manifestations deep sadness, lack of concentration, indecision and the desire to do nothing. It is an illness that affects multiple levels of life, including sports. In this sense, several studies in the field of sports have analyzed the effect of stress and depression in athletes, showing that both factors have been positively associated with chronic diseases [42], dropout [43], suicide [44] and poor sports performance [45]. Due to these negative effects that anxiety, depression, and stress have on the overall health and well-being of athletes, current studies that have linked these mental disorders to eating habits are currently scarce. In addition, this study aims to shed light on the effects that depression and anxiety have on the dietary health of athletes.

### 1.4. Diet

A healthy and balanced diet promotes sports performance and recovery after exercise [46,47]. However, it is necessary to select appropriate foods and liquids and to choose the time of their intake, as athletes need to consume energy during periods of high demand (e.g., training or competition) to maintain body weight, health and maximize performance [46]. Conversely, unbalanced, minimal or poorly recommended food intake may result in decreased muscle mass, loss of bone density, risk of fatigue, injury, illness or a prolonged recovery process and therefore a period away from competition [46].

The Mediterranean diet is one of the most recommended diets because of its positive effects on health [47]. This diet is related to healthy eating habits, based on a balanced contribution of fish, cereals, olive oil, eggs and dairy products (preferably yoghurt or cheese), fruit and vegetables and a lower consumption of red meat, and refined, ultraprocessed and sweetened products [48]. Different studies have shown that adherence to this type of diet tends to reduce the risk of cardiovascular disease, or incidence of cancer mortality [48]. In this way, the Mediterranean diet presents a healthy pattern in terms of morbidity and mortality. In relation to sport, the Mediterranean diet has been found to be associated with increased sports performance, decreased recovery times from injuries and increased strength in the face of mental illness (eg, depression, eating disorders) [47,49].

### 1.5. Objectives and Hypotheses

Therefore, the present study aims to analyze how the social skills of the trainer influence the psychological well-being, resilience and eating habits of the athlete. To this end, the following hypotheses are put forward: (a) Prosocial skills of the trainer positively predict resilience; anxiety, depression and stress; (b) Antisocial skills negatively predict resilience; (c) Resilience will negatively predict anxiety, depression and stress; (d) Anxiety, depression and stress will negatively predict healthy and balanced eating.

## 2. Method

### 2.1. Participants

This study involved 1547 athletes from various sports disciplines (see Table 1) in southern Spain, 49.97% of whom were men and 50.03% women. The average age of the participants was 28.97 with a standard deviation of 1.98. In addition, 127 trainers participated with an average age of 45.62 years.

The sampling method followed was non-probabilistic incidental, as there were sports clubs that we did not have access to or were not allowed to administer the questionnaires to the athletes.

### 2.2. Measurements

Prosocial and antisocial behavior by trainers. Kavussanu & Boardley’s Prosocial and Antisocial Behavior in Sport Scale (PABSS) was used [50]. This scale was validated and adapted to the Spanish sports context by Navarro, Trigueros, Cangas & Aguilar-Parra [51]. The trainers filled in those items of the questionnaire that referred to prosocial (4 items) and antisocial (5 items) behavior towards their athletes. For the present study, the word "teammate" in the original scale was modified from the items to the words “my players”. The athletes responded through a Likert-type scale of between 1 (strongly disagree) and 7 (strongly agree).

Resilience. Vigário, Serpa & Rosado’s [52] scale was used. This scale was validated and adapted to the Spanish sports context by Trigueros, Aguilar, Álvarez, Alcaraz & Rosado [13]. This instrument is made up of 25 items distributed among the factors personal competence and acceptance of oneself and life. The athletes responded through a Likert type scale of between 1 (disagree) and 7 (totally agree).

Anxiety, stress and depression. The Lovibond & Lovibond [53] scale was used, which was validated and adapted to the Spanish context by Bados, Solanas & Andrés [54]. This scale consists of 21 items that are distributed equally among each of the three factors (depression, stress and anxiety). The athletes responded through a Likert-type scale that ranged from 0 (Doesn’t describe anything that happened to me or I felt during the week) to 3 (Yes, this happened to me a lot, or almost always).

Healthy and unhealthy eating habits. The WHO Wold Survey [55] was used, validated and adapted to the Spanish context by Balaguer [56]. In the present study we selected those indices that referred to the consumption of healthy foods (such as fruit, fish, or vegetables) and unhealthy ones (such as sweets, snacks, or pastries) on a weekly basis. Athletes responded using a Likert scale ranging from 1 (rarely) to 4 (every day). For more details on the indices and their validity, see Balaguer [56].

### 2.3. Procedure

At the beginning of the study, the heads of several sports clubs in southern Spain were contacted and the objective of the study was explained to them. After obtaining the authorization from the managers, it was explained to both trainers and their players that they were going to participate in an investigation related to the ability to cope with the stressors and eating behaviors. In addition, it was emphasized that their participation would be anonymous and voluntary, and those who expressed no desire to participate would be excluded from the study without any form of punishment. The completion of the questionnaires was done before the training sessions, in a space designed to reduce environmental distractions. In addition, during the administration of the questionnaires a member of the research group was present while the participants filled out each of the questionnaires, in case any type of doubt arose.

This study respected all the ethical procedures established by the American Psychology Association. In addition, approval was obtained from the research bioethics committee (UALBIO 2019/014) of the University of Almeria.

### 2.4. Data Analysis

To study the relationships between the variables, the mean, standard deviation, bivariate correlations, reliability analysis through Cronbach’s index and a structural equation model (SEM) were analyzed. The statistical programs SPSS-25 (IBM, Armonk, NY, USA) and AMOS-20 (IBM, Armonk, NY, USA) were used.

To analyze the hypothesized model through a SEM, a bootstrapping of 5000 interactions together with a maximum likelihood estimate was used. The estimators were considered robust despite the lack of normality. The cut-off indices of the model tested were the following [57]: χ^2^/df, equal to or less than 3; Comparative Fit Index, Tucker Lewis Index and Incremental Fit Index, equal to or greater than 0.95; RMSEA (Root Mean Square Error of Approximation) plus its 90% confidence interval (CI); equal to or less than 0.06 and the SRMR (Standardized Root Mean Square Residual) equal to or less than 0.08. These cut-off rates are very restrictive when testing very complex models, so they must be taken into account with some caution [58].

## 3. Results

### 3.1. Descriptive Statistics and Reliability Analysis

Table 2 shows the descriptive statistics (mean and standard deviation) and the bivariate correlations (Pearson). In addition, reliability analyses through Cronbach’s α are shown for each of the study variables, with scores above 0.70 [59].

Bivariate correlations have shown that both prosocial behaviors, resilience and healthy food correlate positively with each other. The same is true for antisocial behaviors, anxiety, stress, depression, and unhealthy foods. However, the correlations were negative between both groups.

### 3.2. Structural Equation Modeling

**Figure 1:**
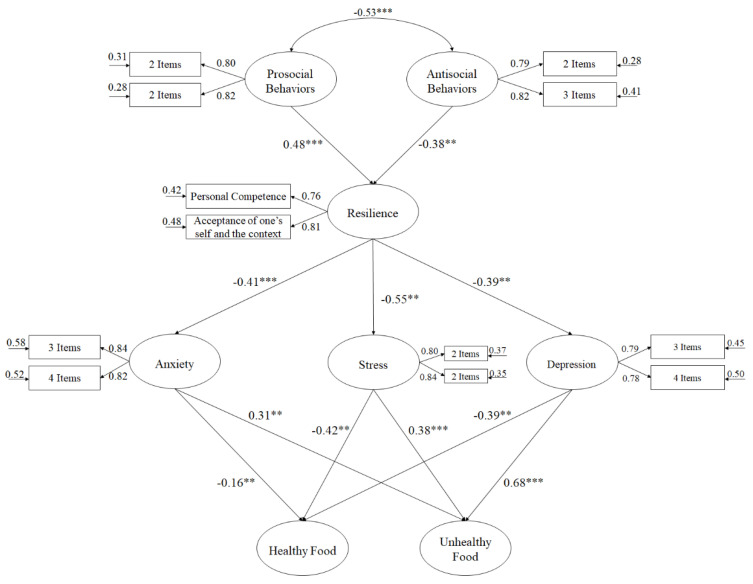
Hypothesized model, analyzed through a structural equation model. Note: *** *p* < 0.001; ** *p* < 0.01.

To analyze the existing relationships, established by the hypothesized model (Figure 1), the number of latent variables was reduced to at least two indicators. This reduction is especially indicated when a complex hypothesized model is presented (see, [60]). The latent variables were: resilience, which included 2 indicators (personal competence and acceptance of oneself and sporting life; [13]). For prososcial behaviours it was necessary to divide the 4 items into 2 indicators, as with the 5 items of antisocial behaviours, the 7 items of stress, depression and anxiety (see, McDonald & Ho [60]).

The results achieved in the model hypothesized (Figure 1) through the SEM, showed acceptable adjustment rates: χ^2^ (67, *N* = 1547) = 195.89, χ^2^/df = 2.92, *p* < 0.001, Incremental Fit Index = 0.96, Comparative Fit Index = 0.96, Tucker Lewis Index = 0.96, RMSEA = 0.049 (CI 90% = 0.042–0.053), SRMR = 0.036. The relationship between the study variables was based on standardized regression weights. 

## 4. Discussion

The present study aimed to analyze how social skills of the trainer influence the resilience, stress, anxiety, depression and eating habits of the athlete. This study looks for the first time at the direct relationship between a trainer’s social skills and his/her players, since previous studies had only analyzed athletes’ perceptions of their trainer. This way, different studies have shown that the perception of athletes about their trainer differs greatly from the opinion that trainers have of themselves [61]. In this sense, the figure of the trainer is considered key for sportsmen and women, since he or she constitutes the main reference point of the team. In addition, this study analyses for the first time a direct relationship between the capacity to face up to stressors, mental health and the relationship with eating habits, habits that are essential for the physical and mental health of the athletes, and for their good performance during competition [6].

The results of the present study have shown how the trainer’s prosocial behaviors were positively related to resilience, while antisocial behaviors were negatively predicted. These results can only be compared, with studies that have analyzed autonomy support and control behaviors, whose meanings are related to prosocial and antisocial behaviors respectively. A study by Trigueros, Aguilar-Parra, Cangas-Díaz, Fernández-Batanero, Mañas, Arias & López-Liria [2], support for autonomy positively predicted resilience, while control behaviors indirectly predicted it negatively. Similarly, a study by Gillham, Gillham, & Hansen [62] showed that athletes’ resilience developed if there is social cohesion and prosocial behaviour among team members, including the trainer. Similarly a study by Lu, Lee, Chang, Chou, Hsu, Lin, & Gill [63] where they analyzed the interactions between trainer and athlete, from the perspective of the players, showed that those athletes who felt respected and involved by the trainer had greater resilience and less stress and anxiety. Thus, these results highlight the importance of the trainer’s social behaviors. In this sense, those trainers who listen carefully to their athletes’ comments, show kindness and understanding, and try to correct and help develop their skills, will tend to develop a greater capacity to adapt to the possible changes and stressors that they have or have to face. On the other hand, if trainers were to insult, threaten and/or punish their athletes when interacting with them, their resilience would be diminished, making them more vulnerable to difficulties and feeling a sense of weariness towards sports practice.

On the other hand, the results showed that resilience was positively related to anxiety, stress and depression. However, there is a large fragmentation in the relationship of resilience with these three variables, with no evidence of studies that have related them. Despite this, a study conducted through interviews by Sarkar & Hilton [64] with Olympic athletes showed that those of them who had high levels of resilience showed greater control of anxiety and were less predisposed to stress during competition. Similarly, a study by Trigueros, Aguilar-Parra, Álvarez, Cangas & López-Liria [65] showed that those athletes who had high levels of resilience were negatively related to anxiety. In addition, those athletes who had high levels of resilience showed less anxiety about injuries and a shorter convalescence time [66]. On the other hand, a study conducted by García-Secades, Molinero, Ruiz-Barquín, Salguero, De la Vega & Márquez [67] on professional athletes showed that those with high levels of resilience negatively predicted stress, increasing their sense of well-being during competition. Similarly, a study by Joyce, Smith & Vitaliano [29] with female athletes showed that those with lower levels of resilience showed a greater predisposition towards mental illness and competitive stress. Finally, there are hardly any studies that have analysed the direct relationship between resilience and depression, however, a study by Drew & Matthews [68] showed that student-athletes were less prone to depression and mental illness if they had high levels of resilience. Thus, the present study shows the need to promote the adaptability and the capacity to overcome the multiple challenges and difficulties that athletes have to face throughout their sporting life. To this end, it is necessary to promote a climate that favours the achievement of objectives, teamwork and optimal challenges [69].

Finally, the results have shown that anxiety, stress and depression have been positively related to the consumption of unhealthy food, and negatively related to healthy food by the athlete. These results are in line with several studies. The study carried out by Lanfranchi, Maïano, Morin & Therme [70], stands out where those young people who practice physical exercise and sport assiduously showed a greater prevalence of suffering from eating disorders when they felt high anxiety. From the field of health, the studies by Oliver, Wardle & Gibson [71] and Wardle, Steptoe, Oliver & Lipsey [72] stand out, where it was observed that those participants who were subjected to stressful situations showed a greater predilection for foods with more sugar and calorie content than those participants who were more relaxed. Similarly, a study conducted by Sánchez-Villegas, Toledo, De Irala, Ruiz-Canela, Pla-Vidal & Martínez-González [73] showed that those people diagnosed with depression showed a greater predilection for eating fast food and ultraprocessed foods, which also caused a false sense of emotional well-being which in turn led to an increase in depression. In this way, the results of this study show that athletes can experience moments in their sporting life where they feel psychologically and emotionally more fragile, experiencing anxiety, stress and depression. This situation can cause changes in the behaviour and habits of athletes, such as in their diet, which are contrary to their physical and personal well-being and quality of life. Therefore, it is essential to have psychological intervention programs aimed at prevention when athletes manifest symptoms such as depression and stress. In this sense, programs such as mindfulness or emotional intelligence have obtained good results in multiple contexts (e.g., education, health, work), including sports [74,75,76] when dealing with complex or negative emotional situations.

Despite the results achieved in this study, it is necessary to highlight the existence of a series of limitations. Among them, it should be noted that the selection of the sample has followed a non-probabilistic, incidental procedure. Moreover, it is a relational study and therefore does not allow the establishment of causal relationships, so that the relationships between the variables can have multiple interpretations. Likewise, the study was based on the use of self-reported questionnaires. Finally, future research should analyse the influence of mindfulness-based therapies on eating disorders in athletes, in addition to analysing the impact of the social context on the mental health of athletes.

## 5. Conclusions

Results have shown that the trainer’s prosocial behaviors are positively related to resilience, while antisocial behaviors are negatively related to resilience. Resilience was negatively related to anxiety, stress and depression. Finally, anxiety, stress and depression were negatively related to healthy eating and positively related to unhealthy eating. In short, these results show the importance of trainers interacting with their athletes in a respectful and understanding way, since it will have a series of positive consequences not only for the psychological and emotional well-being of the athlete but also to develop his/her self-esteem and self-confidence, being able to face the stressors.

## Figures and Tables

**Table 1:** Distribution of the sample by sport discipline.

Sports Discipline	Sport Men	Sport Women	Trainers
Football	234	187	25
Basketball	201	167	38
Voleyball	198	234	40
Handball	140	186	24
*N* =	1547		127

**Table 2:** Descriptive statistics, bivariate correlation and reliability analyses.

Factors	M	SD	α	1	2	3	4	5	6	7	8
1. Prosocial Behaviors	4.78	0.78	0.86	-	−0.62 ***	0.57 **	−0.42 ***	−0.45 ***	−0.57 ***	0.22 *	−0.12 *
2. Antisocial Behaviors	1.42	1.29	0.82		-	−0.41 **	0.53 ***	0.60 **	0.58 ***	−0.15 *	0.13 *
3. Resilience	4.92	1.63	0.82			-	−0.48 **	−0.35 ***	−0.67 **	0.52 **	−0.26 **
4. Anxiety	1.12	0.99	0.87				-	0.20 ***	0.45 ***	−0.33 **	0.61 ***
5. Stress	1.08	0.77	0.81					-	0.78 **	−0.28 ***	0.44 **
6. Depression	0.98	0.86	0.86						-	−0.46 ***	0.66 ***
7. Healthy Food	3.02	0.77	0.78							-	−0.58 ***
8. Unhealthy Food	1.97	1.03	0.82								-

Note: *** *p* < 0.001; ** *p* < 0.01; * *p* < 0.05.
